# Superior calyceal access vs. other calyceal access in percutaneous nephrolithotomy: A systematic review and meta-analysis

**DOI:** 10.3389/fsurg.2022.930159

**Published:** 2022-09-13

**Authors:** Yucheng Ma, Lede Lin, Zhumei Luo, Tao Jin

**Affiliations:** ^1^ Department of Urology, Institute of Urology (Laboratory of Reconstructive Urology), West China Hospital, Sichuan University, Chengdu, China; ^2^ Department of Oncology, Chengdu Third People’s Hospital, Chengdu, Sichuan, China

**Keywords:** percutaneous nephrolithotomy, superior calyceal access, meta-analysis, stone clearance, complication

## Abstract

**Purpose:**

To evaluate the superior calyceal access’s performance and safety in relation to other calyceal access during percutaneous nephrolithotomy (PCNL).

**Methods:**

The suggested reporting items for systematic reviews and meta-analysis were used to conduct this meta-analysis (PRISMA). To find pertinent studies for this meta-analysis, we searched PubMed, Embase, Web of Science, and China National Knowledge Infrastructure (CNKI). Operation time and hospital stay are two secondary outcomes, whereas failed stone clearance and complication events are the two major outcomes. Utilizing Stata 15.0, RevMan 5.3, and R 4.0.2, relative data were extracted and evaluated.

**Results:**

This meta-analysis was based on 16 studies with 8,541 individuals. Pooled results suggested that superior calyceal access could offer fewer failed stone clearance [odds ratio (OR): 0.64, 95% confidence interval (CI), 0.47–0.88, *P* = 0.006] and lower additional puncture rate (OR: 0.35, 95% CI, 0.24–0.51, *P* < 0.001) than other calyceal access. No complication difference was found (OR: 1.10, 95% CI, 0.78–1.56, *P* = 0.57). Superior calyceal access could offer shorter operation time [standard mean difference (SMD): −0.57, 95% CI, −0.98 to −0.15, *P* = 0.007]. No hospital stay difference was found (SMD: 0.07, 95% CI, −0.09 to 0.22, *P* = 0.38). Large heterogeneity was detected in stone clearance comparison (*I*^2 ^= 71%, *P* < 0.001) and operation time (*I*^2 ^= 97%, *P* < 0.001). The stone clearance comparison also identified significant publication bias (*P* = 0.026). These defects weaken the credibility of the results.

**Conclusion:**

Superior calyceal access in PCNL may result in a higher stone clearance rate, a lower rate of subsequent punctures, and a faster operation duration with no increase in postoperative complications or hospital stay for kidney stone patients, despite the significant heterogeneity and publication bias. By conducting bigger randomized controlled studies, this discovery still has to be confirmed.

## Introduction

Kidney stones are a common disease in the urology department, with prevalence rates of 1%–20% (1). With the development of surgical technology, percutaneous nephrolithotomy (PCNL), a less invasive procedure, has emerged as the preferred method of treating urinary calculi (2). Research on PCNL has always focused on selecting the best renal calyces to create a PCNL channel. This topic was supported by a sizable multicenter prospective study that was published in 2012. Superior calyceal access was found to have a higher potential for problems, longer hospital stays, and a poorer rate of stone clearance than inferior calyceal access (3). It is important to note that the experimental and control groups in this study had significantly different stone locations. A randomized controlled clinical experiment, however, produced findings that diverged from those of earlier research (4). Superior calyceal access had a higher stone clearance rate and a similar postoperative complication rate while treating lower calyx and renal pelvis stones in this randomized controlled trial (RCT) compared to inferior calyceal access. With the exception of the RCT and representative large-sample multicenter prospective cohort study (PCS), many other published studies’ conclusions are inconsistent (5, 6). We conducted this meta-analysis to combine and examine published data in order to produce a greater degree of evidence due to the inconsistency of earlier studies on this subject.

## Method

### Literature search and inclusion criteria

This meta-analysis was carried out according to preferred reporting items for systematic reviews and meta-analysis (PRISMA). We searched PubMed, Embase, Web of Science, and China National Knowledge Infrastructure (CNKI) to identify relevant studies. The latest search date was December 1, 2021. The searching keywords included percutaneous nephrolithotomy, PCNL, superior calyx, upper calyx, superior calyceal access, and upper calyceal access. Furthermore, the reference part of every candidate literature study was manually screened to find possible data sources.

Detailed inclusion criteria followed PICO principles:

1. Patient: Patients with kidney stones were treated with percutaneous nephrolithotomy.
2. Intervention: Intervention in this analysis was superior calyceal access.
3. Comparison: The comparison was conducted between superior calyceal access and other calyceal access.
4. Outcomes: The primary outcomes were stone clearance and complication events. Other outcomes, such as operation and hospital stay time, were not compulsory.

Two independent authors did all the title screening, abstract screening, and full-text review (YM and LL). Exclusion criteria were as follows: reviews, meta-analysis, letters, comments, case serials, and conference abstracts were excluded. Studies focused on comparing middle and inferior calyceal access or published earlier than 2000 were excluded. Studies that did not offer enough information or data which could be used for meta-analysis were excluded.

Nonrandomized primary articles were evaluated by the Newcastle-Ottawa Scale (NOS) system, and two independent reviewers performed the evaluation procedure. According to the NOS scales, 7–9 score studies were considered high-level quality, 5–6 score studies were considered moderate-level, and <5 score studies were low-level quality. Low-level quality studies should not be involved in the meta-analysis.

### Meta-analysis

This study compared the efficacy and safety of superior calyceal access applied in the percutaneous nephrolithotomy. In efficacy comparison, the primary outcome was the postoperative stone clearance for two types of access. However, since some included studies did not offer detailed data about complications in the safety comparison, only the overall complication rate was compared in this analysis. Data on the failed stone clearance event, the number of patients with postoperative complications, and the total number of patients were extracted from the included studies. The operation and hospital stay time were also extracted from included studies to compare superior calyceal access and other calyceal access.

Two authors performed the data extracting procedures and double-checked them independently (YM and LL). The data pooling procedures were performed with RevMan 5.3, Stata 15.0, and R 4.0.2. Statistical significance was defined as *P* < 0.05. The primary outcomes’ 95% confidential intervals (95% CI) were also provided. Standard mead difference was calculated and synthesized as an estimate for a continuous variable. The odds ratio (OR) was calculated and synthesized as the primary effect size for the discontinuous variable. Heterogeneity was evaluated by *I*^2^ and Q tests. When *I*^2^ > 50%, heterogeneity was considerable, and a random-effects model should be used. Subgroup analyses were conducted to offer more information to identify potential factors contributing to heterogeneity. Forest plots were produced to display the main results. In addition to funnel plots, Egger’s test was used to detect publication bias. Any detected publication bias was reanalyzed using the trim-and-fill method to evaluate the effect of the publication bias on the meta-analysis results.

## Results

**Figure 1:**
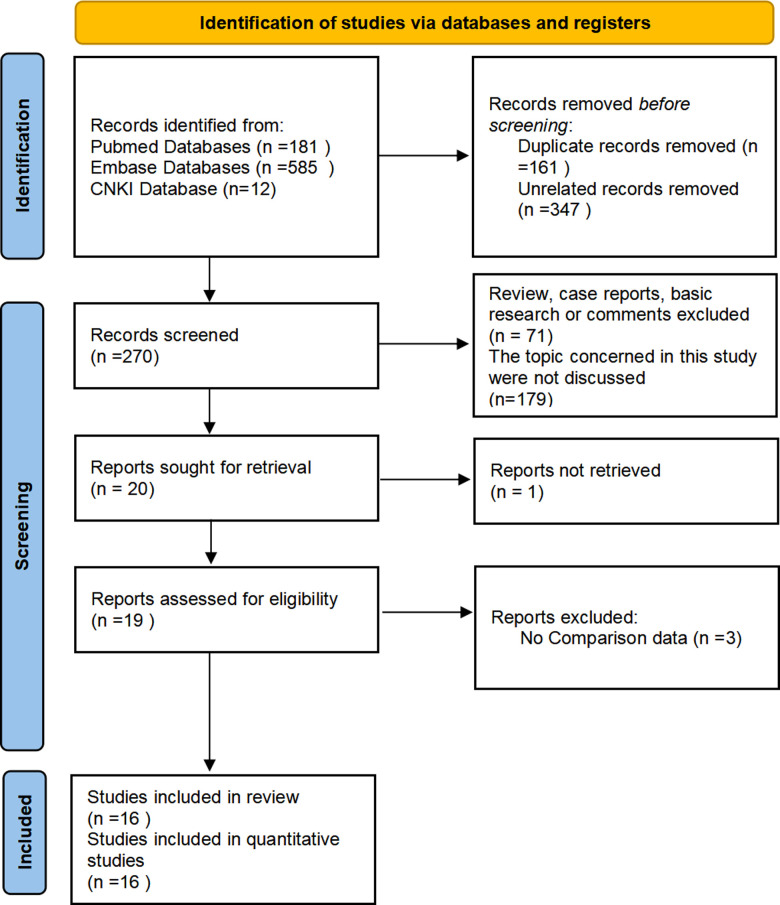
Study searching and screening flow chart.

After database search, 778 studies were identified. Original data from 16 research studies were extracted, and after applying screening techniques and quality assessments, they were included in the quantitative analysis (3–18). The flowchart for screening is shown in Figure 1. The efficacy (failed stone clearance) and safety (complication occurrence) of the superior calyceal access against other calyceal access were compared in 16 research studies (a total of 8,451 patients); additional puncture data were examined in 5 studies (987 participants). As secondary outcomes, the comparison of the operation time included 13 studies, and the comparison of the hospital stay included 8 research studies. One RCT, seven prospective studies, and eight retrospective investigations made up the 16 studies. The common PCNL approach served as the foundation for nearly all of the included investigations. Only one employed both conventional and mini PCNL methods (5). Two studies applied for both the prone and supine postures, and 11 research studies applied for the prone position only. Three further studies failed to mention the patient position in the PCNL. The listed studies’ specifics are provided in Table 1. The included papers did not contain any descriptions of flexible nephoscope application.

**Table 1:** Characteristics of included studies.

Author	Year	study design	Study location	Total Participants	Superior calyceal access participant	Other calyceal location	Other calyceal access participants	Age of included patients	kidney stone location	Patient position	PCNL type	Guided method	Stone clearance definition (residual size)	NOS
Monish Aron	2004	PCS	India	102	69	Inferior calyceal	33	Not reported	Inferior calyceal calculi	Not reported	Standard PCNL	NR	Residual calculi at 1 day (2 mm)	7
Nelson Rodrigues Netto	2004	RCS	Brazil	86	16	Inferior and middle calyceal	70	34 ± 11.88 for superior access; 43.59 ± 11.39 for inferior and middle access	Staghorn stones	Prone position	Standard PCNL	Ultrasonography	Residual calculi at 1 day (5 mm)	6
Ahmet Tefekli	2012	PCS	Multi center	3515	403	Inferior calyceal	3112	Not reported	Upper calyx, middle calyx, lower calyx, renal pelvis, and multiple stones	Both supine or prone position applied	Standard PCNL	X-ray fluoroscopy or ultrasonography	Residual calculi at 1 month (NR)	8
Rohit Singh	2014	PCS	India	94	43	Inferior calyceal	51	39.84 ± 10.42 for superior access; 39.53 ± 10.23 for inferior access	Staghorn stones	Prone position	Standard PCNL	X-ray fluoroscopy	Auxiliary procedures needed	7
Vishwajeet Singh	2015	RCT	India	100	50	Inferior calyceal	50	38.6 ± 6.5for superior access; 38.2 ± 7.7 for inferior access	Inferior calyceal and pelvic stones	Prone position	Standard PCNL	X-ray fluoroscopy	Auxiliary procedures needed	/
Faruk Özgör	2015	RCS	Turkey	360	42	Inferior and middle calyceal	318	46.7 ± 14.1for superior access; 47.5 ± 10.4 for middle access; 44.6 ± 13.6 for inferior access	Isolated lower, middle, or upper calyceal stones	Prone position	Standard PCNL	X-ray fluoroscopy	Residual calculi at 3 month (4 mm)	7
Yan Song	2016	RCS	China	153	45	Inferior and middle calyceal	108	48.9 ± 9.2 for superior access; 50.2 ± 9.6 for middle access; 47.7 ± 5.5 for inferior access	Single pelvic stone.	Not reported	Standard PCNL	Ultrasonography	Residual calculi at 1 day (5 mm)	7
Kyle A. Blum	2018	PCS	USA	76	17	Inferior calyceal	59	55.22 ± 15.31 for superior access; 55.89 ± 14.74 for inferior access	Staghorn stones	Prone position	Standard PCNL	X-ray fluoroscopy	Residual calculi at 1 month (NR)	6
Liquan Zhou	2018	RCS	China	1438	415	Inferior and middle calyceal	1023	46.32 ± 10.31 for superior access; 47.82 ± 11.54 for inferior and middle access	Upper calyx, middle calyx, lower calyx, renal pelvis, and multiple stones	Prone position	Standard PCNL	X-ray fluoroscopy or ultrasonography	Residual calculi at 3 days (4 mm)	6
Sedat Oner	2018	PCS	Turkey	77	10	Inferior and middle calyceal	67	9.58 ± 3.88 for superior access; 12.56 ± 2.97 for inferior and middle access	Upper calyx, middle calyx, lower calyx, renal pelvis, and multiple stones	Prone position	Standard PCNL	X-ray fluoroscopy	Residual calculi at 1 day (4 mm)	7
Ricardo M. O. Soares	2019	RCS	USA	329	227	Inferior and middle calyceal	102	Not reported	Not reported	Prone position	Standard PCNL	X-ray fluoroscopy	Residual calculi at 1 day (3 mm)	7
Patrick L. Vande Lune	2019	RCS	USA	591	424	Inferior and middle calyceal	167	Not reported	Upper calyx, middle calyx, lower calyx, renal pelvis, and multiple stones	Not reported	Standard PCNL	X-ray fluoroscopy	Residual calculi at 1 day (NR)	7
Charles U. Nottingham	2020	PCS	USA	767	112	Inferior and middle calyceal	655	58.35 ± 14.27 for superior access; 56.71 ± 18.45 for middle access; 54.25 ± 17.09 for inferior access	Upper calyx, middle calyx, lower calyx, renal pelvis, and multiple stones	Prone position (mostly prone position, indicated that there might be some other position such as supine)	Standard PCNL	X-ray fluoroscopy	Residual calculi at 1 day (NR)	8
M. Amaresh	2021	PCS	India	126	63	Inferior calyceal	63	45.81 ± 3.72 for superior access; 46.6 ± 3.41 for inferior access	Inferior calyceal and pelvic stones;	Prone position	Standard and mini PCNL	X-ray fluoroscopy	Residual calculi at 1 month (4 mm)	7
Suxi Huang	2021	RCS	China	258	206	Inferior calyceal	52	43.34 ± 8.30 for superior access; 44.46 ± 7.38 for inferior access	Inferior calyceal calculi	Prone position	Standard PCNL	ultrasonography	Residual calculi at 3 day (4 mm)	8
Yu Zhang	2021	RCS	China	379	146	Inferior and middle calyceal	233	52.22 ± 11.33 for superior access; 48.46 ± 10.82 for middle access; 49.42 ± 9.79 for inferior access	Staghorn calculi	Prone position	Standard PCNL	X-ray fluoroscopy	Residual calculi at 1 day (4 mm)	6

PCNL, percutaneous nephrolithotomy; NR, not reported; PCS, prospective cohort study; RCS, retrospective cohort study; RCT, randomized controlled trial; NOS, Newcastle-Ottawa Scale.

**Figure 2:**
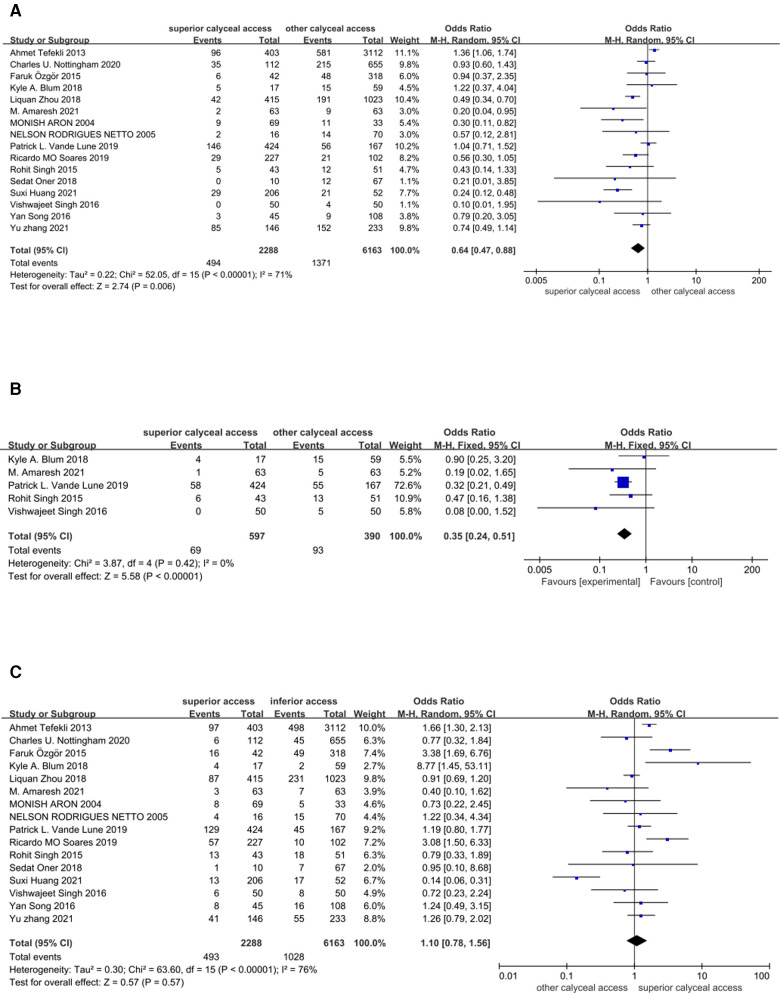
Forest plots of meta-analysis. (**A**) Stone clearance comparison between superior calyceal access and other calyceal access. (**B**) Additional puncture rate comparison between superior calyceal access and other calyceal access. (**C**) Complication comparison between superior calyceal access and other calyceal access.

**Figure 3:**
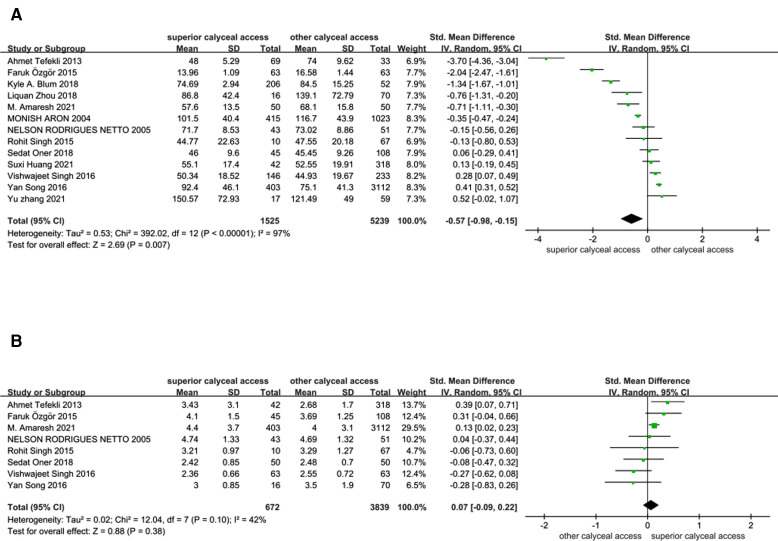
Forest plots of meta-analysis. (**A**) Operation time comparison between superior calyceal access and other calyceal access. (**B**) Hospital stays comparison between superior calyceal access and other calyceal access.

**Figure 4:**
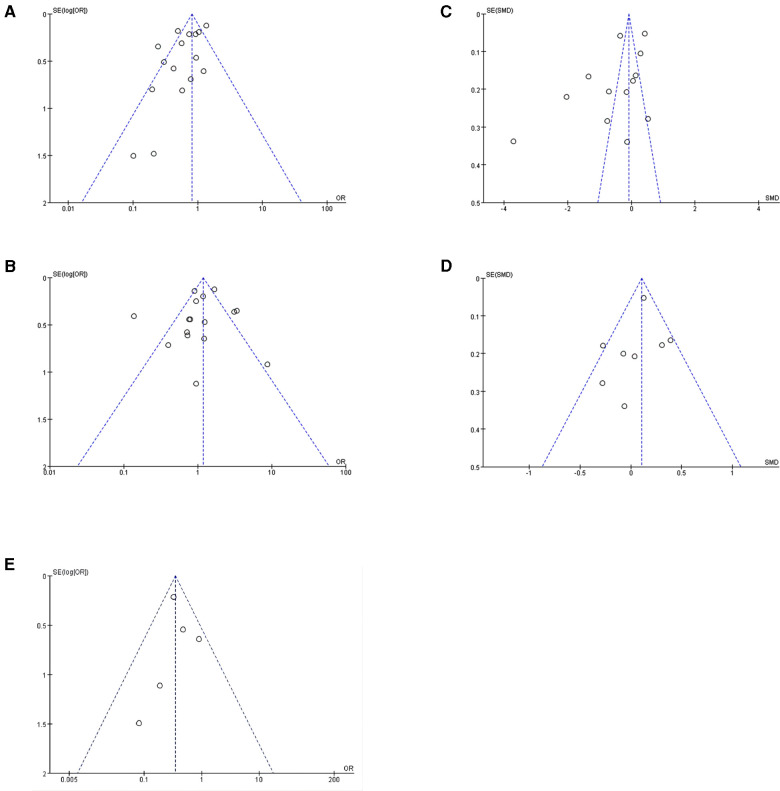
Funnel plots of meta-analysis. (**A**) Funnel plot for stone clearance comparison. (**B**) Funnel plot for complication comparison. (**C**) Funnel plot for operation time comparison. (**D**) Funnel plot for hospital stay comparison. (**E**) Funnel plot for additional puncture comparison.

There were 16 studies (comprising 8,451 participants, 2,288 superior calyceal access and 6,163 other calyceal access) included in the comparison of failed stone clearance (3–18). In the overall synthesis, superior calyceal access could offer less failed stone clearance (higher stone-free rate) than other calyceal access [Mantel–Haenszel statistic (M–H) random model, OR: 0.64, 95% CI, 0.47–0.88, *P* = 0.006, Figure 2A]. The heterogeneity of overall synthesis was significant (*I*^2 ^= 71%, *P* < 0.001). The Egger test (*P* = 0.026) and funnel plot (Figure 3A) detected significant publication bias. The trim-and-fill method was applied to adjust the effect of publication bias on the stability of the results. After adding six additional studies, the pooled result was insignificant (OR: 0.87, 95% CI, 0.60–1.24, *P* = 0.437, Figure 4). Many interesting things were found in the subgroup analysis (Table 2). Compared with studies in other parts of the world, more positive results have been published in Asia (OR: 0.47, 95% CI, 0.34–0.66, *P* < 0.001). Unlike prospective studies, retrospectively designed studies support the conclusion that superior calyceal access can provide better stone clearance than other calyceal access (OR: 0.62, 95% CI, 0.44–0.88, *P* = 0.007).

**Table 2:** Subgroup analyses of additional puncture comparison.

Subgroup	Pooled OR for additional puncture		Heterogeneity	
	OR (95% CI)	*P*-value	*I* ^2^	*P*-value
Recent 5-year study (yes)				
Yes	0.35 (0.24–0.52)	<0.001	23.5%	0.271
No	0.34 (0.13–0.89)	0.028	22.4%	0.256
Study design				
RCT	0.08 (0.00–1.52)	0.093	—	—
PCS	0.51 (0.24–1.09)	0.082	0.0%	0.446
RCS	0.32 (0.21–0.49)	<0.001	—	—
Study location				
Asia	0.30 (0.13–0.73)	0.008	0.0%	0.441
America	0.36 (0.24–0.55)	<0.001	56.3%	0.130
Sample size > 200				
Yes	0.32 (0.21–0.49)	0.018	—	—
No	0.42 (0.21–0.86)	<0.001	5.7%	0.365
Other access type				
Inferior calyceal access	0.42 (0.21–0.86)	<0.001	5.7%	0.365
Inferior and middle calyceal access	0.32 (0.210.49)	0.018		
Types of kidney stones				
Staghorn calculi	0.62 (0.27–1.40)	0.246	0.0%	0.446
Not specified	0.30 (0.20–0.45)	<0.001	0.0%	0.588
Types of PCNL				
Standard PCNL	0.36 (0.25–0.52)	<0.001	14.9%	0.318
Standard PCNL + mini PCNL	0.19 (0.02–1.65)	0.131	—	—
Guide method: x-ray fluoroscopy	0.35 (0.24–0.51)	<0.001	0.0%	0.424

OR, odds ratio; CI, confidence interval; RCT, randomized controlled trial; PCS, prospective cohort study; RCS, retrospective cohort study; PCNL, Percutaneous nephrolithotomy.

In this meta-analysis, since many included studies did not report the first puncture success rate, we used additional puncture as another efficacy outcome for data pooling. Five studies offered relative information comprising 597 patients who received superior calyceal access and 390 patients who received other calyceal access (4, 5, 7, 8, 16). In the overall comparison, superior calyceal access could offer a lower multiple puncture rate (OR: 0.35, 95% CI, 0.24–0.51, *P* < 0.001, Figure 2B) without significant heterogeneity detected (*I*^2^ = 0%, *P* = 0.42). No significant publication bias was detected in the funnel plot and Egger test (*P* = 0.663, Figure 3E). In the PCNL conducted on the patients with staghorn stone, there was no significant difference between superior calyceal access and other calyceal access (OR = 0.62, 95% CI, 0.27–1.40, *P* = 0.246, Table 3). In the subgroup analysis, we found that in the prospectively designed studies, the comparison is not significant (PCS: OR = 0.51, 95% CI, 0.24–1.09, *P* = 0.082; RCT: OR = 0.08, 95% CI, 0.00–1.52, *P* = 0.093, Table 3), this result may indicate possible selecting and reporting bias in the retrospectively designed studies included in this meta-analysis.

**Table 3:** Subgroup analyses of failed stone clearance.

Subgroup	Pooled OR for failed stone clearance		Heterogeneity	
	OR (95% CI)	*P*-value	*I* ^2^	*P*-value
Recent 5-year study (yes)				
Yes	0.63 (0.45–0.87)	0.006	61.7%	0.005
No	0.63 (0.31–1.27)	0.197	67.0%	0.010
Study design				
RCT	0.10 (0.01–1.95)	0.129	—	—
PCS	0.70 (0.41–0.18)	0.179	67.5%	0.005
RCS	0.62 (0.44–0.88)	0.007	60.6%	0.013
Study location				
Asia	0.47 (0.34–0.66)	<0.001	34.8%	0.129
America	0.90 (0.70–1.16)	0.411	0.0%	0.504
Sample size >200				
Yes	0.73 (0.50–1.06)	0.094	82.4%	<0.001
No	0.46 (0.28–0.75)	0.002	0.0%	0.497
Other access type				
Inferior calyceal access	0.45 (0.19–1.04)	0.063	83.8%	<0.001
Inferior and middle calyceal access	0.74 (0.58–0.93)	0.010	28.4%	0.192
Types of kidney stones				
Pelvic calculi	0.79 (0.20–3.05)	0.727	—	—
Staghorn calculi	0.72 (0.50–1.05)	0.086	0.0%	0.645
Inferior calyceal calculi	0.26 (0.15–0.45)	<0.001	0.0%	0.728
Not specified	0.75 (0.50–1.10)	0.143	74.7%	<0.001
Types of PCNL				
Standard PCNL	0.67 (0.49–0.92)	0.012	71.3%	<0.001
Standard PCNL + mini PCNL	0.19 (0.04–0.95)	0.043	—	—
Guide method				
X-ray fluoroscopy	0.78 (0.61–1.01)	0.056	19.4%	0.264
Ultrasonography	0.38 (0.18–0.83)	0.015	30.4%	0.238
X-ray fluoroscopy or ultrasonography	0.82 (0.30–2.25)	0.706	95.4%	<0.001
NR	0.30 (0.11–0.82)	0.019	—	—
Clearance definition				
Additional treatment needed	0.36 (0.12–1.03)	0.056	0.0%	0.367
Residuals identified by imaging	0.67 (0.48–0.93)	0.015	73.3%	<0.001

OR, odds ratio; CI, confidence interval; RCT, randomized controlled trial; PCS, prospective cohort study; RCS, retrospective cohort study; PCNL, percutaneous nephrolithotomy; NR, not reported.

There were also 16 studies (comprising 8,451 participants, 2,288 superior calyceal access and 6,163 other calyceal access) included in the complication comparison meta-analysis (3–18). In the overall synthesis, no significant complication difference was detected between superior calyceal access and other calyceal access (M–H random model, OR: 1.10, 95% CI, 0.78–1.56, *P* = 0.57, Figure 2C). The heterogeneity of synthesis was significant (*I*^2 ^= 76%, *P* < 0.001). No publication bias was found by Egger’s test (*P* = 0.568) and funnel plots (Figure 3B). Detailed subgroup analyses about safety information are shown in Table 4, and there was no significant difference in any subgroup.

**Table 4:** Subgroup analyses of complication.

Subgroup	Pooled OR for complication		Heterogeneity	
	OR (95% CI)	*P*-value	*I* ^2^	*P*-value
Recent 5-year study (yes)				
Yes	1.00 (0.62–1.60)	0.983	78.8%	<0.001
No	1.38 (0.86–2.20)	0.184	53.6%	0.056
Study design				
RCT	0.72 (0.23–2.24)	0.566	—	—
PCS	1.09 (0.62–1.89)	0.771	55.7%	0.035
RCS	1.16 (0.69–1.94)	0.581	84.7%	<0.001
Study location				
Asia	0.84 (0.51–1.39)	0.488	76.7%	<0.001
America	1.65 (0.87–3.13)	0.125	64.2%	0.025
Sample size >200				
Yes	1.16 (0.74–1.83)	0.513	87.0%	<0.001
No	0.97 (0.61–1.56)	0.914	16.3%	0.302
Other access type				
Inferior calyceal access	1.38 (0.98–1.93)	0.601	86.2%	<0.001
Inferior and middle calyceal access	0.79 (0.33–1.91)	0.066	60.2%	0.010
Types of kidney stones				
Pelvic calculi	1.24 (0.49–3.15)	0.647	—	—
Staghorn calculi	1.35 (0.71–2.58)	0.361	45.9%	0.136
Inferior calyceal calculi	0.30 (0.06–1.54)	0.150	80.5%	0.023
Not specified	1.32 (0.92–1.91)	0.133	71.9%	<0.001
Types of PCNL				
Standard PCNL	1.15 (0.81–1.63)	0.432	77.1%	<0.001
Standard PCNL + mini PCNL	0.40 (0.10–1.62)	0.200	—	—
Guide method				
X-ray fluoroscopy	1.37 (0.90–2.10)	0.148	61.9%	0.005
Ultrasonography	0.57 (0.12–2.75)	0.486	86.9%	<0.001
X-ray fluoroscopy or ultrasonography	1.23 (0.68–2.23)	0.487	90.1%	0.001
NR	0.73 (0.22–2.45)	0.615	—	—

OR, odds ratio; CI, confidence interval; RCT, randomized controlled trial; PCS, prospective cohort study; RCS, retrospective cohort study; PCNL, percutaneous nephrolithotomy; NR, not reported.

**Figure 5:**
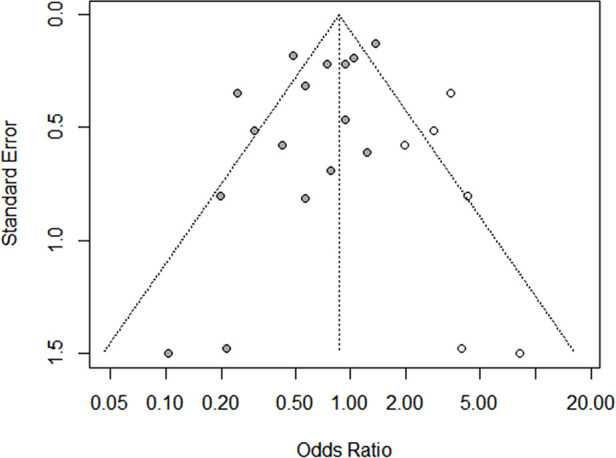
Funnel plot for trim-and-fill analysis. Hollow circle means added simulated studies.

In this analysis, operation time and hospital stay were synthesized and compared as secondary outcomes. Since there might be differences in operation time and hospital stay definition among included studies, standard mean difference (SMD) was calculated and pooled to get a proper estimate. There were 13 studies (1,525 superior calyceal access and 5,239 other calyceal access) included in the operation time comparison (3–13, 17, 18). After the data synthesis, we found that superior calyceal access could offer a short operation time compared with other calyceal access [inverse variance weighted method (IVM), SMD: −0.57, 95% CI, −0.98, −0.15, *P* = 0.007, Figure 5A]. The heterogeneity was substantial (*I*^2 ^= 97%, *P* < 0.001). The funnel plot was asymmetric, but Egger’s test detected no publication bias (*P* = 0.074) (Figure 3C). Hospital stay comparison was conducted among eight studies (672 superior calyceal access, 3,839 other calyceal access) (3–7, 12, 13, 17), and we found that there was no significant difference (IVM, SMD: 0.07, 95% CI, −0.09, 0.22, *P* = 0.38, Figure 5B) without significant heterogeneity (*I*^2 ^= 42%, *P* = 0.10). No publication bias was detected by funnel plot (Figure 3D) and Egger’s test (*P* = 0.408).

## Discussion

The choice of access for PCNL is crucial and has given rise to a heated discussion. Superior calyceal access may provide a higher stone clearance rate than other calyceal access, according the findings of the meta-analysis (M–H random model, OR: 0.64, 95% CI, 0.47–0.88, *P* = 0.006). This finding is, however, undermined by significant heterogeneity and substantial publication bias. The difference in stone clearance between superior and other calyceal access became minor when publication bias was adjusted using the trim-and-fill technique (six studies included, OR: 0.87, 95% CI, 0.60–1.24, *P* = 0.437). When comparing additional puncture rates, we discovered that superior calyceal access might provide a lower additional puncture rate than other calyceal access (OR: 0.35, 95% CI, 0.24–0.51, *P*0.001) without a lot of heterogeneity. There was no discernible difference between superior and inferior calyceal access in the safety comparison (OR: 1.08, 95% CI, 0.76–1.53, *P* = 0.68). However, significant heterogeneity and possible publication bias continued to have an impact on this conclusion. The combination of operation time and hospital stay was a secondary outcome in this analysis. Superior calyceal access and other calyceal access did not significantly shorten hospital stays (IVM, SMD: 0.07, 95% CI, −0.09, 0.22, *P* = 0.38, Figure 2D). Similar to stone clearance, despite significant variations in the length of the operation (IVM, SMD: −0.57, 95% CI, −0.98, −0.15, *P* = 0.007, Figure 2C), the significant heterogeneity has a major impact on the stability of the conclusion (*I*^2^ = 97%, *P*0.001).

Many considerations, including but not limited to the location and size of the stone, additional patient problems like stone burden, location, pelvicalyceal anatomy, history of prior surgery, and the surgeon’s technical preferences, influence the surgeon’s choice of access site (3). A prior study found that with superior calyceal access for patients with superior calyceal and staghorn stones, PCNL might be a preferable option. Its primary benefit is anatomical closeness and instrumentation simplicity (3). Numerous other findings concurred that difficult kidney stones should benefit from greater utilization of superior calyceal access (8, 9). One funnel-shaped calyx that discharges the superior calyx is a distinguishing feature of the superior calyceal access, and the internal space is often bigger than that of the inferior and middle calyces (19). The superior renal calyx has a considerably lower likelihood of developing massive calculi than the middle and inferior renal calyx due to the gravity effect, which is also highly helpful for increasing the operational area and flexibility of the device inside the kidney (20). In addition to facilitating access to the renal pelvis and other calices and achieving a satisfactory stone-free rate with fewer punctures and fewer complications, access through the superior calyx also enables the device to be aligned with the long axis of the kidney in comparison to other access points (8). Although some studies believed that the middle calyceal access could treat both superior and inferior calyx (21), the passage through the middle calyx forms a very small acute angle with the superior calyx and inferior calyx. It was often challenging to perform thorough stone cleaning (20). The advantages of the superior calyx are the main drawbacks of PCNL’s use of the inferior calyx access. The inferior calyx has a more intricate structure, a smaller internal volume, and a larger angle with the kidney’s long axis; all of these characteristics may have an impact on the clearance rate for stones (20). Unless there is clear evidence that superior calyceal access carries a higher risk of perioperative adverse events, such as superior calyceal placement to the 11th and 12th intercostals, the choice of inferior calyceal access and superior calyceal access is more dependent on the doctor’s habits and even the hospital’s tradition (3).

The main difference between superior and inferior calyceal access in terms of problems is that establishing the upper calyx channel frequently necessitates supracostal puncture, which may increase the risk of chest-related complications like pneumothorax and even lung injury (5). The risk of hydrothorax following supracostal puncture was minimal (3.31%), which was within an acceptable range, according to a study that was specifically focused on this procedure (22). Superior calyceal access has been linked to higher difficulties in some research, but this study incorporated more published data and discovered that it does not in overall comparison and subgroup analysis. This may be due to the fact that the researchers in the original studies that made up this meta-analysis excluded obese patients from the superior calyceal access for ethical and medical grounds when the superior calyceal position was too high in such patients. We suspect that selection bias may have a bigger impact on the complication comparison because the majority of the original research in our investigation used unblinded designs, nonrandomized prospective trials, and retrospective studies. Postoperative problems can arise during surgery for various reasons, including stone burden, stone placement, pelvicalyceal anatomy, baseline features, patient posture (prone or supine), tract size, and scope type. Although the results of this study, which included 16 original studies and 8,451 patients’ postoperative data, indicated that statistically superior calyceal access may not increase the risk of complications, it is still necessary to consider the actual patient characteristics when selecting the puncture technique.

An excellent systematic review related to this topic was published in 2019 (23). The authors of that comprehensive review evaluated supracostal and infracostal access for percutaneous nephrolithotomy and came to the conclusion that while supracostal access was safe, infracostal access PCNL was more successful. This conclusion differs from those observed in this study since there was no discernible difference in the rates of complications between superior calyceal access and other calyceal access. This distinction also has an explanation. First of all, unlike the goals of our investigation, the primary objective of the previously published meta-analysis was not to compare supracostal access with infracostal access. Second, there was no discernible difference between infracostal and supracostal access in the entire comparison.

To sum up, we believe that, if safety is completely taken into account prior to surgery, superior calyceal access may be able to result in improved stone free rate (SFR) and a quicker postoperative recovery period in PCNL surgery. A thoroughly planned randomized clinical trial must be conducted to confirm this conclusion.

There were still some limitations of this analysis. First, although the major component studies were prospective designed studies, there was only one RCT included in this meta-analysis. Second, although the pooling result indicated that superior calyceal access could offer a better stone clearance rate, the heterogeneity and publication bias weaken the evidence. Third, although many published research studies included comparisons of data between various PCNL approaches, some did not compare the results primarily based on calyceal access, which inherently creates bias. Notably, when planning a calyx approach in PCNL surgery, urologists must take into account various patient-related aspects. Stone burden, location, pelvic architecture, and history of surgery were among them. Patients with greater suprarenal calyces, especially above the 11th or 12th rib, were likely to experience more perioperative difficulties when they have a superior calyceal access. Only after carefully documenting and examining the aforementioned criteria can meaningful inferences be made. Although the original research included in this analysis attempted to adequately gather the patient’s baseline data, none of the studies were able to produce a thorough report on the pertinent baseline characteristics. Therefore, all conclusions of this study need to be treated with caution. In the future, more large-scale randomized studies should be focused on this topic.

## Conclusion

Superior calyceal access in PCNL may result in improved stone clearance rate, lower additional puncture rate, faster operation time, and no increase in postoperative complications or hospital stay for kidney stone patients, despite the significant heterogeneity and publication bias. Larger randomized controlled studies still need to be conducted in order to confirm this finding.

## Data Availability

The original contributions presented in the study are included in the article/Supplementary Material, further inquiries can be directed to the corresponding author.
